# Refining Drug Classes: A Multidimensional, Data-Driven Map of Antidepressant Pharmacology

**DOI:** 10.1016/j.bpsgos.2026.100796

**Published:** 2026-07-24

**Authors:** Daniel Martins, Lia Fernandes, Steven C.R. Williams, Federico Turkheimer, Mitul A. Mehta

**Affiliations:** a RISE-Health, Department of Clinical Neurosciences and Mental Health, Faculty of Medicine, University of Porto, Porto, Portugal; b Department of Neuroimaging, Institute of Psychiatry, Psychology and Neuroscience, King’s College London, London, United Kingdom; c Institute for Human and Synthetic Minds, King’s College London, London, United Kingdom

**Keywords:** Antidepressants, Data-driven classification, Neuromodulatory systems, Polypharmacology, Receptor-binding affinity

## Abstract

**Background:**

Traditional classifications of antidepressant medications are organized around primary molecular targets or historical development pathways. However, these categorical systems obscure substantial heterogeneity in receptor- and transporter-level pharmacology both within and across classes. Many antidepressants exhibit clinically relevant polypharmacology, engaging multiple neuromodulatory systems at biologically meaningful affinities. Therefore, we undertook a data-driven reappraisal of antidepressant pharmacology based on multidimensional ligand–target binding profiles.

**Methods:**

Experimentally measured inhibition constants (Ki) were curated from public pharmacological databases to construct receptor and transporter affinity “fingerprints” for 25 commonly prescribed antidepressants across serotonergic, dopaminergic, histaminergic, cholinergic, and adrenergic targets. Median pKi values were aggregated to generate a drug × target affinity matrix. This matrix was analyzed using unsupervised hierarchical clustering and principal component analysis, without reference to conventional therapeutic class labels.

**Results:**

Antidepressants formed a structured yet continuous pharmacological landscape organized along graded dimensions reflecting transporter selectivity versus receptor-level polypharmacology. Drugs traditionally grouped within the same class frequently diverged in their multidimensional affinity profiles, whereas compounds from different classes often clustered together. Histaminergic, muscarinic, and adrenergic targets were major contributors to the separation between broadly acting and more selective agents.

**Conclusions:**

Antidepressants are more accurately characterized by multidimensional affinity architectures than by categorical class labels. Affinity-based representations provide a mechanistically transparent framework for linking molecular pharmacology to systems-level brain function and for understanding how antidepressants engage distributed neuromodulatory systems beyond their traditionally defined primary targets.

## Plain language summary

Antidepressants are usually grouped into categories such as SSRIs, SNRIs, and tricyclic antidepressants, but these labels may not fully reflect how the drugs work in the brain. In this study, we analyzed published data on how 25 antidepressants interact with multiple brain targets involved in neurotransmission, including serotonin, noradrenaline, dopamine, histamine, acetylcholine, and adrenergic systems. Using data-driven methods, we created pharmacological fingerprints for each drug and compared their overall binding profiles. We found that antidepressants do not form clear-cut categories; instead, they lie along a continuous spectrum. Drugs within the same class often differed considerably, whereas drugs from different classes sometimes showed similar profiles. Differences in interactions with histamine, cholinergic, and adrenergic systems appeared particularly important and may help explain variation in sleep, alertness, cognition, and side effects. These findings suggest that antidepressants may be better understood through multidimensional pharmacological profiles rather than fixed drug classes, providing a more biologically informed framework for future personalized psychiatry.

Antidepressant medications are commonly grouped into broad pharmacological classes, such as selective serotonin reuptake inhibitors (SSRIs), serotonin and norepinephrine reuptake inhibitors (SNRIs), tricyclic antidepressants, and atypical agents, based on their primary molecular targets or historical development pathways (1, 2, 3). Although clinically convenient, these categorical labels obscure the substantial heterogeneity in receptor- and transporter-level binding profiles that exists both within and across classes (4). Many antidepressants exhibit marked polypharmacology, interacting with multiple neuromodulatory systems at affinities that are likely to be biologically relevant at therapeutic doses (5). As a result, drugs nominally assigned to the same class can differ profoundly in their effects on arousal, cognition, sleep, autonomic function, and side-effect burden (5,6).

This mismatch between traditional classifications and underlying molecular mechanisms has important implications (7,8). Clinically, it limits the ability to predict individual differences in tolerability and symptom response. Conceptually, it constrains efforts to link antidepressant action to systems-level models of brain function that emphasize distributed neuromodulatory control rather than single-target effects (9). Increasingly, it has become clear that antidepressants should be understood not as selective modulators of isolated pathways but as agents that shift the global operating regime of neuromodulatory networks, involving serotonin, norepinephrine, dopamine, histamine, acetylcholine, and adrenergic signaling (10).

These limitations have motivated recent initiatives to move beyond indication- and class-based drug labels toward mechanism-informed frameworks. Notably, the Neuroscience-Based Nomenclature (NbN) proposes classifying psychotropic medications according to their primary pharmacological domains and modes of action, with the aim of improving biological transparency and clinical communication (7,8). Although NbN represents an important conceptual advance, it necessarily relies on predefined target categories and does not explicitly capture the graded, multidimensional nature of receptor- and transporter-level engagement that characterizes many antidepressants.

Large-scale pharmacological databases now provide an opportunity to revisit antidepressant classification from a bottom-up, data-driven perspective. By leveraging experimentally measured binding affinities across multiple molecular targets, it is possible to construct quantitative pharmacological “fingerprints” for individual drugs that capture their multidimensional interaction profiles. Unsupervised analyses of such fingerprints offer a principled way to identify latent structure in antidepressant pharmacology, free from a priori assumptions about drug classes or primary mechanisms of action (11). This strategy has already been successfully applied to antipsychotic drugs, where affinity-based clustering revealed groupings that diverged from traditional typical versus atypical distinctions, instead highlighting gradients of dopaminergic, serotonergic, histaminergic, and cholinergic engagement. These findings demonstrated that receptor-level similarity does not always align with indication-based labels and provided a more mechanistic framework for understanding efficacy, side-effect profiles, and augmentation strategies. Extending this approach to antidepressants enables a comparable reexamination of their pharmacological architecture, potentially uncovering previously unrecognized relationships between molecular profiles, circuit-level effects, and clinical phenotypes.

**Figure 1:**
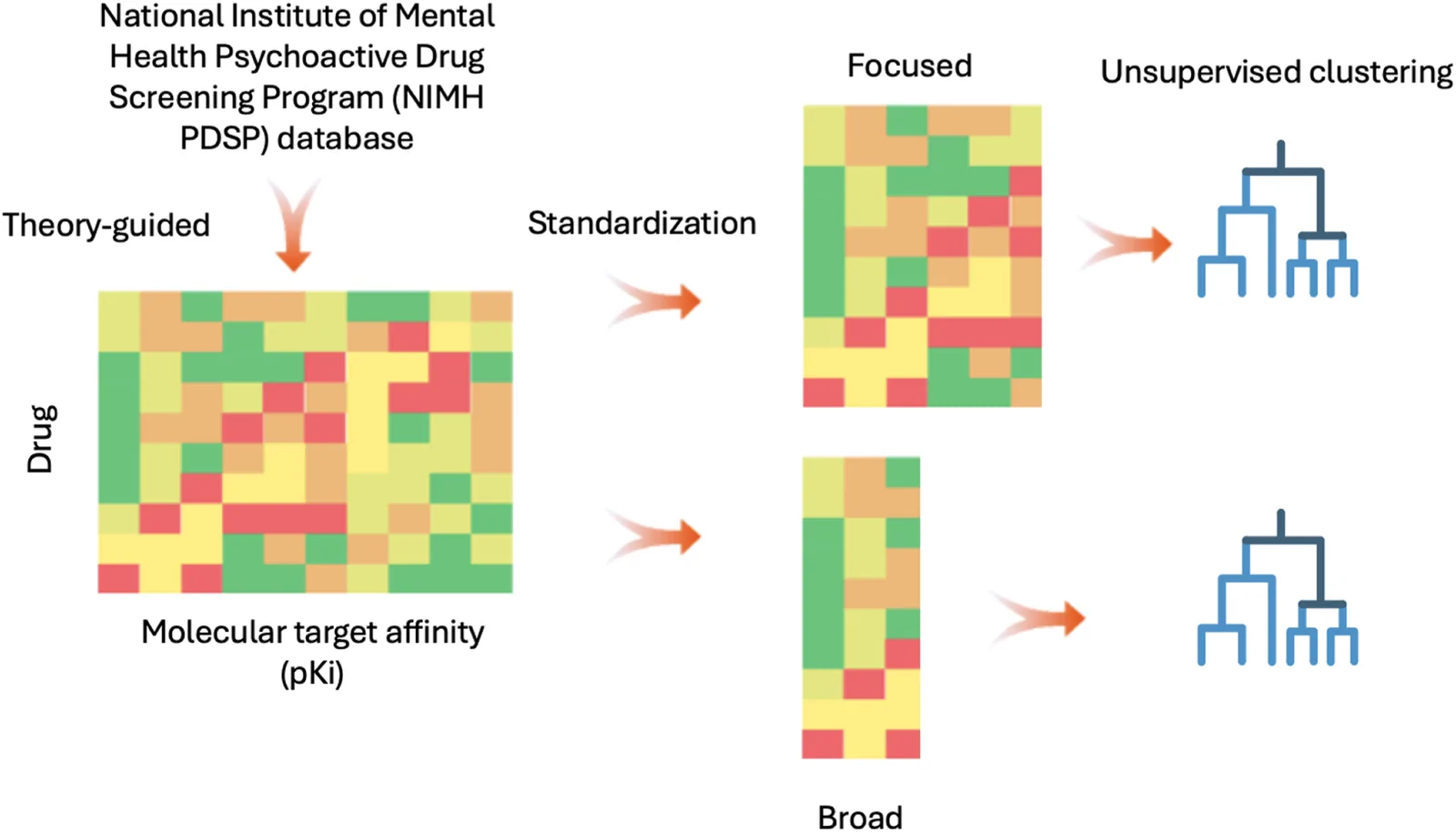
Schematic diagram of the analysis pipeline. Molecular target affinity profiles (pKi) for psychoactive compounds were obtained from the NIMH PDSP database. Drug–target matrices were standardized and analyzed using 2 complementary strategies: a focused approach, restricted to selected targets of theoretical interest, and a broad approach including all available targets. In both cases, standardized affinity profiles were subjected to unsupervised hierarchical clustering to identify data-driven groupings of compounds based on shared pharmacological signatures.

In the present study, we apply this approach to a curated database of ligand–target affinity measurements to derive a data-driven classification of antidepressants based on their receptor and transporter binding profiles. Focusing on neuropharmacologically relevant systems, including monoaminergic, histaminergic, cholinergic, and adrenergic targets, we construct a drug × target affinity matrix using robust aggregation of binding data and examine its structure using unsupervised clustering and dimensionality reduction techniques. Our aim is not to redefine antidepressant efficacy or clinical indications but to provide a mechanistically grounded taxonomy that better reflects the multidimensional nature of antidepressant action and offers a principled framework for interpreting differences in tolerability and side-effect burden among drugs with broadly comparable efficacy, while linking molecular pharmacology to systems-level brain function and individual variability in treatment response. Please see Figure 1 for a summary of our analytic pipeline.

## Methods and Materials

### Data Source and Ligand Selection

Ligand–target affinity data were obtained from a curated Ki database comprising experimentally measured inhibition constants (Ki) for small-molecule ligands across a broad range of neurotransmitter receptors, transporters, and related molecular targets. Only entries reporting numeric Ki values were retained for analysis. To focus on clinically relevant compounds, we restricted the dataset to antidepressant medications currently or historically used in routine psychiatric practice, including SSRIs, SNRIs, tricyclic antidepressants, and multimodal or atypical antidepressants. Ligand names were normalized to account for spelling variants and salt forms before filtering.

Consistent with previous pharmacological mapping approaches (11), receptor and transporter binding affinities were derived from publicly available sources that aggregate experimentally measured Ki values, including the National Institute of Mental Health Psychoactive Drug Screening Program database (https://pdsp.unc.edu/pdspweb/). This strategy enabled the construction of comprehensive ligand–target affinity profiles suitable for cross-drug comparison while remaining agnostic to therapeutic class labels. Only data from studies that reported binding to human tissue were included.

### Affinity Preprocessing and Transformation

Ki values were converted to molar units and transformed to pKi values (pKi = −log_10_ Ki) to linearize the affinity scale and ensure comparability across targets. For ligand–target pairs with multiple reported measurements, pKi values were aggregated using the median, a robust estimator that minimizes the influence of outliers and inter-study variability. Ligand–target pairs with no available affinity data were treated as missing at this stage.

### Construction of the Affinity Matrix

A ligand × target affinity matrix was constructed using median pKi values. To ensure sufficient data density and reduce noise from sparsely characterized targets, targets were retained only if affinity data were available for at least 40% of the antidepressants in the sample. Missing values in the retained matrix were imputed with a low-affinity floor (pKi = 4, corresponding to approximately 100 μM) to allow stable multivariate analyses while conservatively representing weak or absent binding.

### Theory-Guided Target Selection

To facilitate mechanistic interpretability, analyses focused on a predefined set of neuropharmacologically relevant targets encompassing monoaminergic, histaminergic, cholinergic, and adrenergic systems. This included serotonin receptors and transporters, dopamine receptors and transporters, the norepinephrine transporter, histamine H_1_ receptors, muscarinic acetylcholine receptors, and α- and β-adrenergic receptors. This selection reflects established mechanisms underlying antidepressant efficacy and side-effect profiles, while remaining agnostic to traditional drug class labels.

### Broad and Focused Pharmacological Analyses

To characterize the structure of antidepressant pharmacology at different levels of resolution, we performed 2 complementary analyses: a broad (coverage-driven) analysis and a focused (theory-guided) analysis. Both analyses used the same preprocessing pipeline, affinity transformation, standardization procedures, and unsupervised methods, differing only in target selection strategy and coverage thresholds.

In the broad analysis, target selection was driven by data availability rather than biological specificity. Only molecular targets with high coverage across antidepressants were retained (affinity data available for ≥60% of drugs), resulting in a reduced set of well-sampled receptors and transporters. This approach yielded a compact, low-sparsity affinity matrix that provides an agnostic overview of antidepressant pharmacology, emphasizing robust similarities and differences based on consistently characterized molecular interactions.

In the focused analysis, target selection was guided by neuropharmacological relevance. We restricted the analysis to receptors and transporters belonging to serotonergic, noradrenergic, dopaminergic, histaminergic, cholinergic, and adrenergic systems, which are implicated in antidepressant action, arousal regulation, cognition, and side-effect profiles. To retain mechanistically important targets that are less consistently assayed across compounds, a lower coverage threshold (≥40%) was applied, yielding a richer and more detailed representation of antidepressant polypharmacology across core neuromodulatory systems.

Comparing results across broad and focused analyses allowed us to assess the robustness of the pharmacological structure under different constraints while distinguishing patterns driven by high-confidence, widely sampled targets from those emerging when biologically meaningful but less densely characterized interactions are included.

### Standardization

Prior to multivariate analysis, pKi values were *z* scored across drugs for each target (target-wise standardization). This procedure ensured that clustering and dimensionality reduction were driven by relative pharmacological profiles rather than absolute differences in binding magnitude across targets.

### Unsupervised Clustering

Data-driven classification of antidepressants was performed using hierarchical clustering on the standardized affinity matrix. Pairwise distances between drugs were computed using correlation distance, capturing similarity in affinity profiles independent of overall binding strength. Clustering was performed using average linkage. Cluster membership was determined by cutting the dendrogram at a fixed number of clusters (*k* = 5), chosen to balance interpretability and granularity. Cluster assignments were used solely for descriptive purposes and not treated as ground-truth categories.

### Dimensionality Reduction

To visualize the low-dimensional structure of antidepressant affinity profiles, principal component analysis (PCA) was applied to the standardized affinity matrix. The first 2 PCs were retained for visualization, and the proportion of variance explained by each component was reported. PCA was used as an exploratory tool to aid interpretation of continuous pharmacological gradients rather than as a classifier.

## Results

### Antidepressant Affinity Matrix and Target Coverage

After filtering and preprocessing, the final dataset comprised 25 antidepressant medications with affinity data across a focused set of neuropharmacologically relevant targets. Following coverage-based filtering, 29 targets spanning serotonergic, dopaminergic, noradrenergic, histaminergic, cholinergic, and adrenergic systems were retained, with a median target coverage of approximately 60% across drugs. Median pKi values were used to construct a drug × target affinity matrix, capturing the multidimensional binding profiles of individual antidepressants.

### Data-Driven Clustering Reveals Distinct Pharmacological Profiles

**Figure 2:**
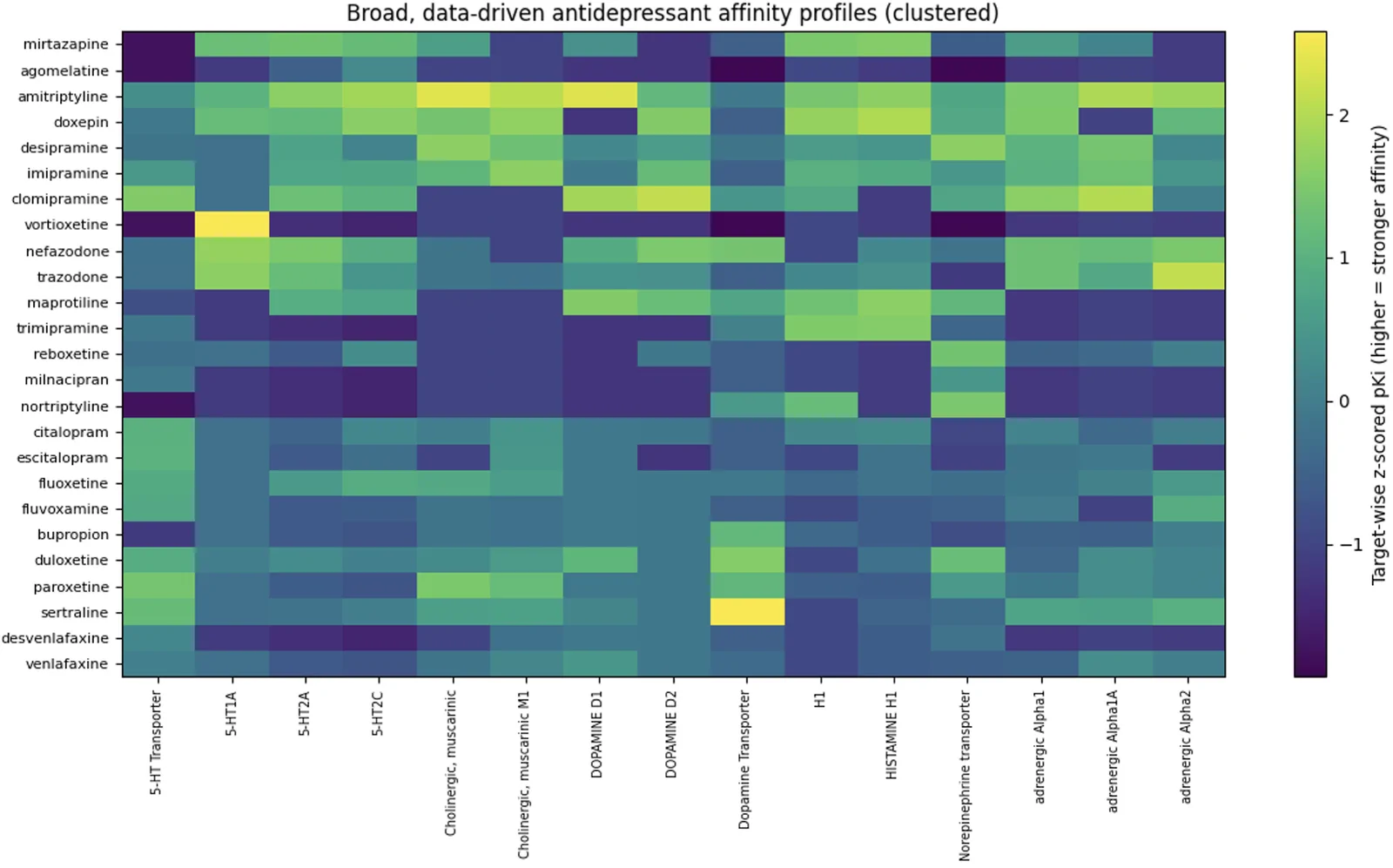
Broad, data-driven mapping of antidepressant receptor and transporter affinity profiles. Clustered heatmap showing standardized binding affinities (target-wise *z* scored pKi) for 25 antidepressant medications across molecular targets meeting high coverage criteria. Rows represent drugs and columns represent receptors and transporters spanning serotonergic, dopaminergic, noradrenergic, histaminergic, cholinergic, and adrenergic systems. Drugs are ordered according to unsupervised hierarchical clustering based on correlation distance of affinity profiles, highlighting structured similarity that cuts across conventional antidepressant class labels. Color intensity reflects relative affinity within each target (yellow = stronger relative affinity; purple = weaker relative affinity). This agnostic analysis reveals heterogeneity within traditional drug classes and convergence between drugs historically assigned to different classes, supporting a multidimensional organization of antidepressant pharmacology. 5-HT, serotonin.

**Figure 3:**
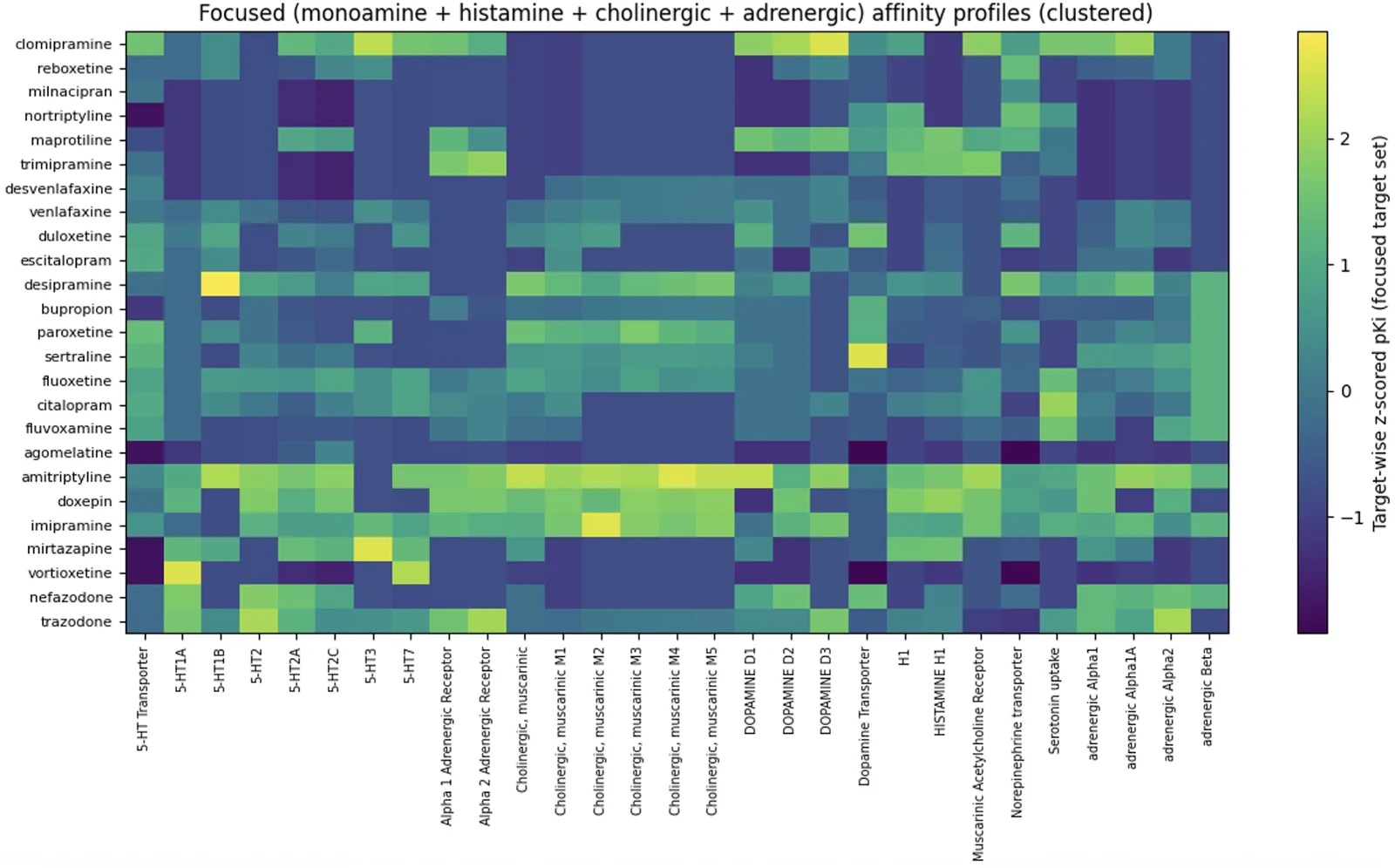
Focused analysis of antidepressant affinity profiles across core neuromodulatory systems. Clustered heatmap showing standardized binding affinities (target-wise *z* scored pKi) for antidepressant medications across a theory-guided set of neuropharmacologically relevant targets, including serotonergic, dopaminergic, noradrenergic, histaminergic, cholinergic, and adrenergic receptors and transporters. Targets were retained using a lower coverage threshold to preserve mechanistically important but less consistently assayed interactions. Drugs are ordered by unsupervised hierarchical clustering based on correlation distance, revealing graded differences in transporter selectivity and receptor-level polypharmacology within and across conventional antidepressant classes. Color intensity reflects relative affinity within each target (yellow = stronger relative affinity; purple = weaker relative affinity), facilitating mechanistic interpretation of pharmacological profiles linked to arousal, cognition, and autonomic regulation. 5-HT, serotonin.

**Figure 4:**
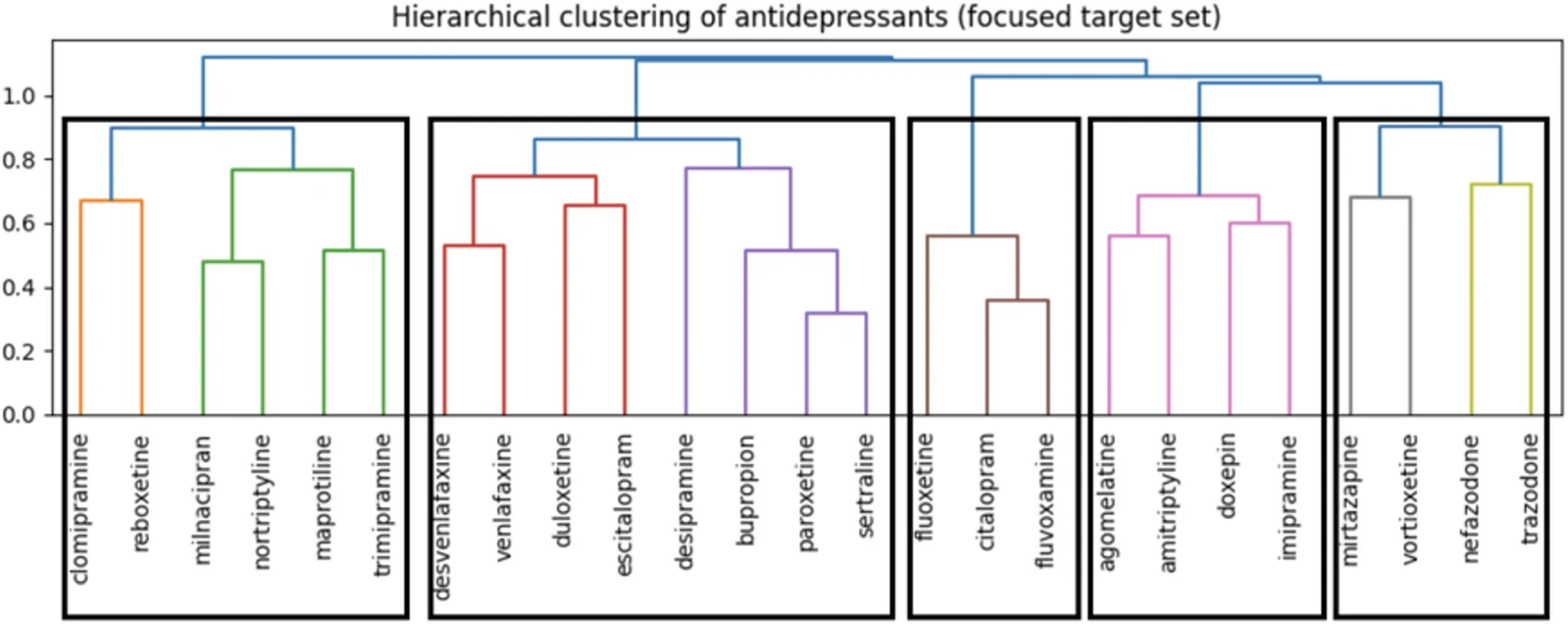
Hierarchical clustering of antidepressants based on focused neuromodulatory affinity profiles and alignment with comparative clinical outcomes. Top panel shows unsupervised hierarchical clustering of antidepressant medications based on standardized binding affinity profiles across a focused, theory-guided set of neuropharmacologically relevant targets, including serotonergic, noradrenergic, dopaminergic, histaminergic, cholinergic, and adrenergic receptors and transporters. Pairwise similarity was computed using correlation distance on target-wise *z-*scored pKi values, and clustering was performed using average linkage. Drugs are labeled along the x-axis and colored by cluster membership (*k* = 5). The resulting structure reveals graded pharmacological similarity that cuts across conventional antidepressant class labels, with transporter-selective agents, broadly polypharmacological compounds, and mixed-profile drugs occupying distinct but partially overlapping branches of the hierarchy.

**Figure 5:**
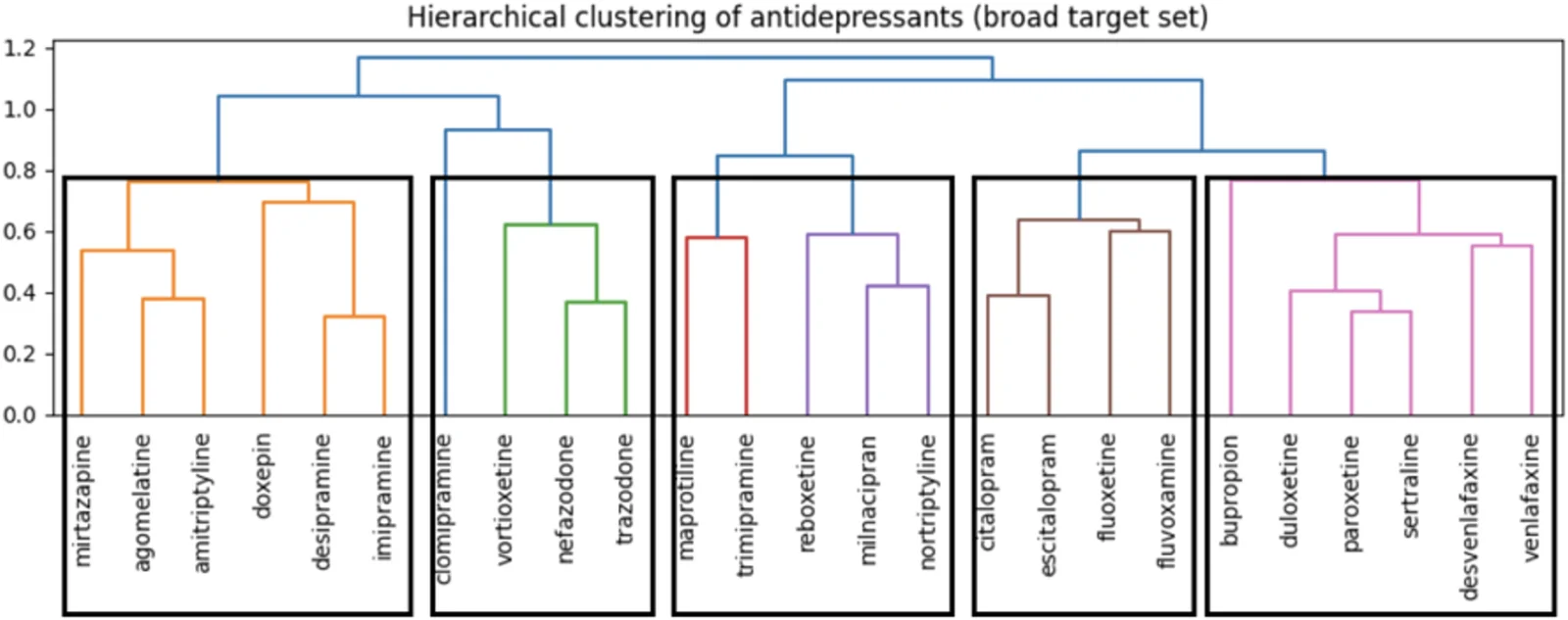
Hierarchical clustering of antidepressants based on broad neuromodulatory affinity profiles and alignment with comparative clinical outcomes. Top panel shows unsupervised hierarchical clustering of antidepressant medications based on standardized binding affinity profiles across a broad, theory-guided set of neuropharmacologically relevant targets, including serotonergic, noradrenergic, dopaminergic, histaminergic, cholinergic, and adrenergic receptors and transporters. Pairwise similarity was computed using correlation distance on target-wise *z-*scored pKi values, and clustering was performed using average linkage. Drugs are labeled along the x-axis and colored by cluster membership (*k* = 5). The resulting structure reveals graded pharmacological similarity that cuts across conventional antidepressant class labels, with transporter-selective agents, broadly polypharmacological compounds, and mixed-profile drugs occupying distinct but partially overlapping branches of the hierarchy.

Unsupervised hierarchical clustering of the standardized affinity matrix identified a clear structure in antidepressant pharmacology that cut across conventional class labels of drugs. Cutting the dendrogram at 5 clusters yielded groups characterized by distinct patterns of receptor and transporter engagement rather than by nominal therapeutic class (Figure 2, Figure 3, Figure 4, Figure 5).

One cluster was dominated by compounds with relatively selective and strong affinity for the serotonin transporter and limited engagement with histaminergic, cholinergic, or adrenergic receptors, largely corresponding to canonical SSRIs. A second cluster comprised agents with combined serotonergic and noradrenergic transporter affinities, including several SNRIs; however, this cluster also included drugs that were traditionally classified outside this group. A third cluster was characterized by broad polypharmacology, including high affinity for muscarinic, histamine H_1_, and α-adrenergic receptors, encompassing several tricyclic antidepressants and related compounds. Additional clusters captured drugs with prominent receptor-level antagonism (e.g., serotonergic and adrenergic receptors) despite relatively weaker transporter binding, as well as agents with mixed or intermediate profiles.

Importantly, drugs assigned to the same traditional class were frequently distributed across different clusters, whereas drugs from different classes often clustered together, indicating that receptor-level affinity profiles do not map neatly onto conventional antidepressant taxonomies.

### PCA Identifies Continuous Pharmacological Gradients

PCA of the standardized affinity matrix further supported a dimensional rather than categorical organization of antidepressant pharmacology. The first 2 PCs together explained a substantial proportion of variance in affinity profiles (>50%). Visualization of drugs in this reduced space revealed continuous gradients rather than discrete groupings, with separation driven by differential engagement of transporter versus receptor targets and by the degree of polypharmacology.

Drugs with high affinity for monoamine transporters and relatively limited off-target binding tended to cluster at one end of the primary axis, whereas drugs with broader receptor engagement, particularly involving histaminergic, muscarinic, and adrenergic systems, occupied opposing regions of the space. Notably, several antidepressants traditionally grouped together occupied widely separated positions in the PCA space, underscoring heterogeneity within established classes.

### Concordance Between Clustering and Dimensional Structure

Cluster assignments derived from hierarchical clustering were broadly consistent with the low-dimensional structure revealed by PCA, with drugs assigned to the same cluster tending to occupy neighboring regions of the PCA space. However, boundaries between clusters were not sharp, reflecting graded transitions in affinity profiles rather than discrete pharmacological categories. Together, these findings indicate that antidepressants are better described by multidimensional affinity fingerprints organized along continuous axes than by a small number of mutually exclusive classes.

## Discussion

In this study, we applied a data-driven analysis of ligand–target affinity profiles to derive a mechanistically grounded classification of antidepressant medications. Rather than relying on traditional therapeutic labels, we characterized antidepressants according to their multidimensional engagement with neurotransmitter receptors and transporters spanning monoaminergic, histaminergic, cholinergic, and adrenergic systems. This approach revealed a structured yet continuous pharmacological landscape in which conventional drug classes were only weakly aligned with underlying molecular profiles.

A central finding is that antidepressant pharmacology is organized along graded dimensions rather than discrete categories. Both hierarchical clustering and PCA converged on the same conclusion: antidepressants differ systematically in the balance between transporter selectivity and receptor-level polypharmacology, and in the extent to which they engage neuromodulatory systems beyond serotonin and norepinephrine. These gradients were not artifacts of sparse data or analytic choices, but they emerged robustly from affinity profiles aggregated across multiple experimental sources. Therefore, drugs traditionally grouped together, such as SSRIs or tricyclic antidepressants, often occupied divergent positions in the pharmacological space, whereas drugs from different historical classes sometimes clustered closely.

This dimensional organization parallels recent data-driven efforts to reclassify psychotropic medications based on receptor affinity profiles rather than historical or clinical labels (7,8,11,12). A receptor-based taxonomy of antipsychotic medications has shown that conventional distinctions such as typical versus atypical antipsychotics fail to capture the underlying pharmacological structure of these compounds (11). Our findings extend this paradigm to antidepressants, demonstrating that similar limitations apply to widely used antidepressant class labels. Together, these convergent results across distinct drug classes support a broader shift in psychopharmacology toward affinity-based representations that reflect how medications engage distributed neuromodulatory systems, rather than privileging a single canonical mechanism of action (7,8).

Although some of the pharmacological gradients identified here—particularly the association between broader receptor-level polypharmacology and reduced tolerability—may align with existing clinical intuition, the present findings provide a systematic and quantitative formalization of these relationships across antidepressants within a unified multidimensional framework. Therefore, the principal contribution of this work is not the identification of a single unexpected clinical effect, but rather the demonstration that antidepressants are more coherently organized by continuous affinity-defined pharmacological architectures than by conventional categorical classes. Such representations may offer a more mechanistically transparent basis for integrating molecular pharmacology with systems neuroscience models, side-effect dimensions, and future approaches to biologically informed treatment stratification. Importantly, several of the tolerability-related gradients identified in the present analysis are broadly consistent with existing psychopharmacological and clinical experience. In particular, the association between broader receptor-level polypharmacology—especially involving histaminergic and muscarinic systems—and reduced acceptability aligns with longstanding clinical observations regarding sedation, anticholinergic burden, autonomic side effects, and treatment discontinuation. The present findings should, therefore, not be interpreted as identifying entirely novel clinical phenomena, but rather as providing a systematic and quantitative framework for formalizing these multidimensional pharmacological relationships across antidepressants within a unified affinity-based representation.

Although the present framework moves beyond traditional antidepressant class labels, it remains situated primarily within the pharmacological space defined by classical monoaminergic systems. The target selection strategy focused on serotonergic, dopaminergic, histaminergic, cholinergic, and adrenergic receptors and transporters, reflecting both the dominant mechanisms of currently prescribed antidepressants and the availability of systematically comparable affinity data across compounds. As such, the present work should not be interpreted as a biologically comprehensive taxonomy of antidepressant action. Rather, it represents a quantitative refinement and reorganization of traditional antidepressant pharmacology within a multidimensional affinity-based framework. Extending this approach to incorporate glutamatergic systems, receptor functional selectivity, intracellular signaling pathways, and network-level pharmacodynamic effects will be an important direction for future work, particularly as newer therapeutic paradigms such as NMDA receptor antagonists and psychedelic compounds become increasingly relevant to antidepressant treatment models.

This reorganization has direct conceptual implications for how antidepressant effects are understood. Many clinically relevant drug properties, such as sedation, cognitive slowing, autonomic side effects, and effects on sleep and arousal, are difficult to reconcile with models that privilege a single primary target (13,14). The present findings instead support a view in which antidepressants act by shifting the global operating regime of neuromodulatory systems, with different drugs producing distinct configurations of receptor and transporter engagement. In this framework, therapeutic and adverse effects emerge from the combined modulation of multiple systems rather than from isolated molecular interactions (12,15).

The prominent contribution of histaminergic and cholinergic targets to the observed pharmacological structure is particularly noteworthy (16). Affinity for histamine H_1_ and muscarinic receptors was a defining feature of drugs with broad polypharmacology, distinguishing them from more transporter-selective agents (17,18). These targets are well known to influence arousal, vigilance, sleep–wake regulation, and cognitive flexibility, suggesting that variation along these dimensions may help explain clinically observed differences in sedative burden, tolerability, and cognitive side effects among antidepressants (17, 18, 19). Importantly, these effects are not adequately captured by conventional antidepressant classifications, yet they emerge naturally when drugs are organized according to receptor-level affinity profiles.

At the same time, the dimensional structure identified here cautions against replacing one rigid taxonomy with another. Although hierarchical clustering provided a useful descriptive summary, cluster boundaries were inherently fuzzy, and many drugs occupied intermediate positions in the affinity space (20). This reinforces the idea that antidepressant pharmacology is best conceptualized in terms of continuous profiles or pharmacological fingerprints rather than mutually exclusive categories. Such representations are better suited to integration with systems neuroscience models, computational frameworks of neuromodulation, and emerging approaches to precision psychiatry that aim to match drug profiles to individual symptom dimensions or vulnerability patterns (21,22). It is important to recognize that many of the pharmacological distinctions highlighted by the present framework are already incorporated implicitly into experienced clinical psychopharmacological practice. Clinicians frequently select antidepressants based not only on formal class assignment, but also on expected sedation, anticholinergic burden, autonomic effects, sleep-related properties, and activation potential, all of which are influenced by receptor-binding profiles beyond primary monoamine transporter targets. Accordingly, the present framework should not be interpreted as replacing established clinical judgment or as providing an immediately actionable precision prescribing tool. Rather, its potential value lies in offering a systematic and quantitative representation of multidimensional pharmacological relationships that are often applied heuristically in practice. Such representations may facilitate mechanistic comparison across drugs, support hypothesis generation, and provide a foundation for future integration with neurobiological, computational, and individual-level clinical data.

Several limitations should be acknowledged. First, the analysis was based on in vitro binding affinity data, which do not directly capture pharmacokinetic factors, active metabolites, receptor occupancy in vivo, or state-dependent neuromodulatory dynamics. As such, the affinity profiles derived here should be interpreted as approximations of molecular interaction potential rather than direct measures of in vivo drug action. In addition, although missing affinity values were imputed using a conservative low-affinity floor to enable stable multivariate analysis, this approach assumes weak or absent binding and may not fully reflect true pharmacological properties in cases of systematically missing data. These considerations may introduce uncertainty in the precise positioning of some compounds within the pharmacological space and should be considered when interpreting cluster structure. Second, although we applied conservative filtering and robust aggregation, affinity data remains heterogeneous in experimental conditions and sources. Third, certain pharmacologically relevant targets were incompletely represented or difficult to capture in a standardized manner. In particular, glutamatergic mechanisms were inconsistently reported across databases, limiting our ability to fully characterize multidimensional pharmacological profiles. Future studies incorporating more comprehensive receptor panels, individual-level clinical data, and longitudinal outcomes will be required to establish the translational relevance of specific pharmacological dimensions more directly. Finally, the present analysis focused on monoaminergic antidepressants, reflecting both their continued centrality in clinical practice and the availability of systematically comparable ligand–target affinity data across these compounds. Extending this framework to include nonmonoaminergic agents, such as NMDA receptor antagonists (e.g., esketamine) or serotonergic psychedelics, represents an important direction for future work. However, these compounds operate through partially distinct pharmacological mechanisms, including glutamatergic modulation, receptor functional selectivity, and complex downstream network effects, which are not fully captured by static binding affinity measures. Incorporating such agents into a unified framework will likely require expansion beyond affinity-based representations to include functional and systems-level metrics. As such, the present study provides a foundation for future efforts to develop more comprehensive, multimodal models of antidepressant pharmacology spanning both conventional and emerging therapeutic mechanisms.

Rather than providing immediate prescriptive guidance, the present framework is best understood as a platform for hypothesis generation. For example, affinity-defined pharmacological dimensions could be related to specific symptom domains, such as sleep disturbance, cognitive impairment, or autonomic dysregulation, enabling more targeted investigation of drug–symptom matching. Similarly, mapping drugs within a shared pharmacological space may support the development of rational polypharmacy strategies by identifying complementary or redundant receptor profiles. Future work integrating affinity-based representations with neuroimaging, electrophysiological, and clinical outcome data will be critical to establishing the translational relevance of these dimensions at the individual patient level. Integrating molecular affinity profiles with systems-level measures may help clarify how different antidepressants influence distributed neural circuits and brain states, thereby advancing mechanistic models of antidepressant action.

### Conclusions

This study demonstrates that antidepressant medications can be meaningfully organized according to their multidimensional receptor and transporter affinity profiles rather than by traditional therapeutic class labels. Using a data-driven analysis of binding affinities across neuropharmacologically relevant targets, we show that antidepressants occupy a structured but continuous pharmacological space, characterized by graded differences in transporter selectivity and receptor-level polypharmacology. These patterns cut across conventional classifications and reveal heterogeneity within established drug classes that are not captured by current nomenclature. These findings support the value of dimensional, affinity-based representations as complementary frameworks for organizing antidepressant pharmacology and generating mechanistically informed hypotheses.

Rather than defining new categories of drugs, this framework emphasizes pharmacological fingerprints that reflect how individual compounds differentially engage serotonergic, noradrenergic, dopaminergic, histaminergic, cholinergic, and adrenergic systems. Therefore, the present framework should be viewed primarily as a descriptive and organizational model of antidepressant pharmacology rather than as an immediately predictive framework for treatment selection or precision prescribing. More broadly, this work supports a shift away from single-target explanations of antidepressant action toward models that recognize the distributed, integrative nature of neuromodulatory control in the brain. Affinity-based representations of drug action offer a principled basis for linking molecular pharmacology to brain state modulation, tolerability profiles, and potentially individual variability in treatment response. As such, they may help inform future efforts in precision psychiatry, where matching drugs to patients requires moving beyond categorical labels toward quantitatively defined, mechanistically grounded profiles.

## Supplementary Material

Key Resource Table

## References

[1] Stahl S.M. (1998). Mechanism of action of serotonin selective reuptake inhibitors. Serotonin receptors and pathways mediate therapeutic effects and side effects. J Affect Disord.

[2] Kline N.S. (1956). Chemotherapy in psychiatry; recent advances. Semin Int.

[3] Marti-Ibanez F., Sackler A.M., Sackler M.D., Sackler R.R. (1956). The challenge of bio- and chemotherapy in psychiatry. J Clin Exp Psychopathol.

[4] Richelson E. (2013). Multi-modality: A new approach for the treatment of major depressive disorder. Int J Neuropsychopharmacol.

[5] Roth B.L., Sheffler D.J., Kroeze W.K. (2004). Magic shotguns versus magic bullets: Selectively non-selective drugs for mood disorders and schizophrenia. Nat Rev Drug Discov.

[6] Montgomery S.A., Kasper S. (1995). Comparison of compliance between serotonin reuptake inhibitors and tricyclic antidepressants: A meta-analysis. Int Clin Psychopharmacol.

[7] Zemach S., Zohar J. (2025). The importance of proper naming—A review of Neuroscience-based Nomenclature (NbN). Asian J Psychiatr.

[8] Zohar J., Stahl S., Moller H.J., Blier P., Kupfer D., Yamawaki S. (2015). A review of the current nomenclature for psychotropic agents and an introduction to the Neuroscience-based Nomenclature. Eur Neuropsychopharmacol.

[9] Hyman S.E. (2012). Revolution stalled. Sci Transl Med.

[10] Taylor C., Fricker A.D., Devi L.A., Gomes I. (2005). Mechanisms of action of antidepressants: From neurotransmitter systems to signaling pathways. Cell Signal.

[11] McCutcheon R.A., Harrison P.J., Howes O.D., McGuire P.K., Taylor D.M., Pillinger T. (2023). Data-driven taxonomy for antipsychotic medication: A new classification system. Biol Psychiatry.

[12] Hopkins A.L. (2008). Network pharmacology: The next paradigm in drug discovery. Nat Chem Biol.

[13] Kelly K., Posternak M., Alpert J.E. (2008). Toward achieving optimal response: Understanding and managing antidepressant side effects. Dial Clin Neurosci.

[14] Gillman P.K. (2007). Tricyclic antidepressant pharmacology and therapeutic drug interactions updated. Br J Pharmacol.

[15] Marder E. (2012). Neuromodulation of neuronal circuits: Back to the future. Neuron.

[16] Owens M.J., Morgan W.N., Plott S.J., Nemeroff C.B. (1997). Neurotransmitter receptor and transporter binding profile of antidepressants and their metabolites. J Pharmacol Exp Ther.

[17] Brown R.E., Stevens D.R., Haas H.L. (2001). The physiology of brain histamine. Prog Neurobiol.

[18] Hasselmo M.E., Sarter M. (2011). Modes and models of forebrain cholinergic neuromodulation of cognition. Neuropsychopharmacology.

[19] Wichniak A., Wierzbicka A., Jernajczyk W. (2012). Sleep and antidepressant treatment. Curr Pharm Des.

[20] Anglhuber C., Edel C., Pimentel E.C.G., Emmerling R., Götz K.U., Thaller G. (2026). Use of cluster analysis for identifying metafounders. J Anim Breed Genet.

[21] Marquand A.F., Rezek I., Buitelaar J., Beckmann C.F. (2016). Understanding heterogeneity in clinical cohorts using normative models: Beyond case-control studies. Biol Psychiatry.

[22] Bzdok D., Meyer-Lindenberg A. (2018). Machine learning for precision psychiatry: Opportunities and challenges. Biol Psychiatry Cogn Neurosci Neuroimaging.

